# 2-Oxoglutarate: linking TCA cycle function with amino acid, glucosinolate, flavonoid, alkaloid, and gibberellin biosynthesis

**DOI:** 10.3389/fpls.2014.00552

**Published:** 2014-10-15

**Authors:** Wagner L. Araújo, Auxiliadora O. Martins, Alisdair R. Fernie, Takayuki Tohge

**Affiliations:** ^1^Departamento de Biologia Vegetal, Universidade Federal de ViçosaViçosa, Brazil; ^2^Max-Planck Partner Group at the Departamento de Biologia Vegetal, Universidade Federal de ViçosaViçosa, Brazil; ^3^Max-Planck-Institut für Molekular PflanzenphysiologiePotsdam-Golm, Germany

**Keywords:** dioxygenases, 2-oxoglutarate dependent dioxygenases, gibberellin, TCA cycle, flavonoid, alkaloid

## Abstract

The tricarboxylic acid (TCA) cycle intermediate 2-oxoglutarate (2-OG) is used as an obligatory substrate in a range of oxidative reactions catalyzed by 2-OG-dependent dioxygenases. These enzymes are widespread in nature being involved in several important biochemical processes. We have recently demonstrated that tomato plants in which the TCA cycle enzyme 2-OG dehydrogenase (2-ODD) was antisense inhibited were characterized by early senescence and modified fruit ripening associated with differences in the levels of bioactive gibberellin (GA). Accordingly, there is now compelling evidence that the TCA cycle plays an important role in modulating the rate of flux from 2-OG to amino acid metabolism. Here we discuss recent advances in the biochemistry and molecular biology of 2-OG metabolism occurring in different biological systems indicating the importance of 2-OG and 2-OG dependent dioxygenases not only in glucosinolate, flavonoid and alkaloid metabolism but also in GA and amino acid metabolism. We additionally summarize recent findings regarding the impact of modification of 2-OG metabolism on biosynthetic pathways involving 2-ODDs.

## INTRODUCTION

**FIGURE 1:**
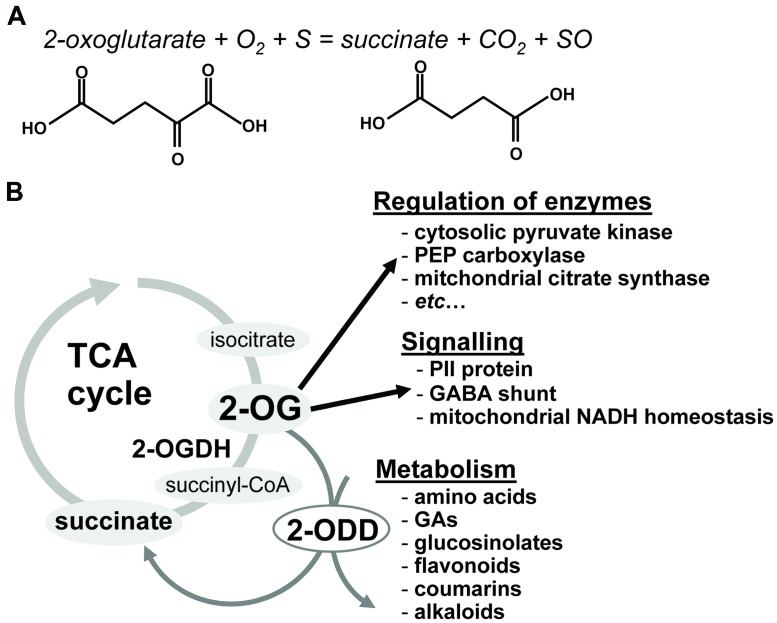
**The general reaction mechanism catalyzed by a 2-oxoglutarate-dependent dioxygenase (2-ODD; **A**) and a schematic overview of reaction and function of 2-ODD in plant (B).** (S) is a substrate and (SO) is the hydroxylated product. 2-OG, 2-oxoglutarate; OGDH, 2-oxoglutarate dehydrogenase, GA, gibberellins; 2-ODD, 2-oxoglutarate-dependent dioxygenase.

2-Oxoglutarate (2-OG), a key organic acid of the tricarboxylic acid (TCA) cycle (Lancien et al., 2000; Scheible et al., 2000), is also an obligatory substrate for 2-OG-dependent dioxygenases (2-ODDs), as depicted in Reaction 1. Briefly, dioxygenases can be defined as enzymes catalyzing reactions in which both atoms of molecular oxygen are incorporated into substrates (**Figure 1**). In the hydroxylation reaction catalyzed by dioxygenases, one atom of molecular oxygen is incorporated into the substrate, while the other atom of oxygen is incorporated into 2-OG resulting in the subsequent formation of succinate and the release of carbon dioxide.

The 2-ODDs are considered the largest known family of non-heme oxidizing enzymes (Prescott and John, 1996; Ozer and Bruick, 2007; Kawai et al., 2014). Members of this family are found throughout biology catalyzing a number of oxidation reactions and have been identified in many organisms ranging from prokaryotes to eukaryotes. Furthermore, oxidative reactions catalyzed by 2-ODD are involved in biosynthetic processes leading to materials of medicinal or agrochemical importance including collagen or other modified polypeptides and amino acids, plant secondary metabolites, phytohormones such as ethylene and gibberellins (GAs) as well as β-lactam antibiotics, i.e., penicillins and cephalosporins (Vaillancourt et al., 2006; Loenarz and Schofield, 2008; Martens et al., 2010 and references therein). Altogether this indicates that 2-ODD and its substrate, 2-OG, are highly important in plant metabolism as a whole and thus a massive impact of their genetic modification on plant metabolism in different plant tissues is expected. It is worth mentioning that a difficulty impeding metabolic engineering within this enzyme family is the fact that multiple pathways within the metabolic network could be affected, linking the enzymes to the specific target metabolites.

Here we discuss recent advances in the biochemistry and molecular biology of 2-OG metabolism occurring in different biological systems indicating the importance of 2-OG and 2-ODDs not only in glucosinolate, flavonoid, and alkaloid metabolism but also in GA and amino acid metabolism.

## THE IMPORTANCE OF 2-OXOGLUTARATE METABOLISM IN HIGHER PLANTS

2-Oxoglutarate participates in a range of reactions in distinct plant cell compartments (Weber and Flügge, 2002; Foyer et al., 2003), also being a key metabolite at the crossroads of carbon/nitrogen metabolism as it is required for ammonia assimilation (Hodges, 2002). Despite this fact, it still remains rather unclear where the major site of production of 2-OG resides. This organic acid can be produced from either sugar respiration or amino acid transamination following the concerted action of isocitrate dehydrogenases, aminotransaminases, and glutamate dehydrogenases (Lancien et al., 2000). We have previously demonstrated that the mitochondrial enzyme 2-OG dehydrogenase (OGDH) has an important role in 2-OG production and metabolism, controlling the levels of this important organic acid in plant cells (Araújo et al., 2008, 2012a,b). These results in conjunction with others in which similar reduction in the TCA cycle activity was also demonstrated (Sienkiewicz-Porzucek et al., 2008, 2010) indicates that mitochondrial TCA cycle enzymes contribute considerably to the regulation of nitrogen assimilation in leaves and that a substantial portion of 2-OG production occurs in the mitochondria itself. It is worth mentioning that the exact balance between the use of cytosolic versus mitochondrial routes for synthesis of 2-OG remains rather unknown. Thus the discovery that the carbon for nitrogen assimilation in leaves comes from carbon stored from the previous night (most likely organic acids such as citrate stored in the vacuole and released to the cytosol; Gauthier et al., 2010) raises the possibility that 2-OG for nitrogen assimilation is generated predominantly in the cytosol and therefore mitochondrial enzymes make only a quantitatively minor contribution. It is important to note that 2-OG is also a direct regulator of several enzymes (e.g., cytosolic pyruvate kinase and PEP carboxylase, mitochondrial citrate synthase, and alternative oxidase) associated with sugar and/or organic acid flux and redox control between cytosol and mitochondria (for a review see Hodges, 2002).

In addition to this, 2-OG has itself been suggested to play a role as a signal metabolite in plants (Lancien et al., 2000; Ferrario-Mery et al., 2001; Feria Bourrellier et al., 2009). This role is, however, largely based on analogy to the important role it plays in conjuncture with the plastidial PII protein in plants (Uhrig et al., 2009). Reports to date suggest that whilst PII may regulates a small number of enzyme systems in plants including *N*-acetyl-glutamate kinase (Ferrario-Mery et al., 2006; Feria Bourrellier et al., 2009) and plastidial acetyl-CoA carboxylase (Feria Bourrellier et al., 2010) its role is unlikely to be as pivotal in plants as in non-plant systems(Araujo et al., 2012). That said it is clear that the production of 2-OG in the mitochondria or cytosol is an important determinant of some plastidial activities.

## ON THE CONNECTIONS BETWEEN 2-OXOGLUTARATE METABOLISM AND GIBBERELLIN IN HIGHER PLANTS

Much effort has been expended on elucidating the physiological functions of the various genes regulated by GA (Yamaguchi, 2008). However, studies concerning the associated effects of GA on energy metabolism and growth are rare. This fact notwithstanding, characterization of the pyruvate dehydrogenase kinase 1 (PDK1) has demonstrated that GA modulates the activity of the mitochondrial pyruvate dehydrogenase by regulating *PDK1* expression and controlling growth in rice (Yazaki et al., 2003; Jan et al., 2006). Collectively it also indicates that GA might modify primary metabolism at the entry point of TCA cycle. In addition it has been demonstrated by the overexpression of genes associated with GA biosynthesis or catabolism that GA levels play key roles on transcriptional programs influencing plant growth (Biemelt et al., 2004; Dayan et al., 2010). Furthermore, reduction of TCA cycle enzymatic activity has led to reduction of GA levels in tomato roots (van der Merwe et al., 2009).

In this vein tomato plants with reduced levels of the TCA cycle enzyme 2-OGDH were recently characterized by early leaf senescence and a modified fruit ripening most likely due to differences in the levels of bioactive GAs (Araújo et al., 2012b). Given that the reduction in the activity of this enzyme was associated to a higher impact on respiration rates than observed previously in other TCA enzyme it seems reasonable to suggest that 2-OG might be of critical importance in the regulation of respiration rates in higher plants. It should be mentioned that both the chemical (Araújo et al., 2008) and molecular (Araújo et al., 2012b) inhibition of 2-OGDH was characterized by significant alterations in both sugars and TCA cycle intermediates. Notably, a compensatory augmentation in the flux of the GABA shunt was clearly observed most likely in an attempt to restore the TCA cycle. In addition, the changes in the GABA shunt suggest that its up regulation is needed to maintain succinate supply to the mitochondrial electron transport chain. This finding highlights the metabolic importance of the GABA shunt in plants (Fait et al., 2008) and is similar to findings observed following neuronal OGDH inhibition (Sá Santos et al., 2006; Shi et al., 2009). Remarkably this compensatory up regulation of the GABA shunt was coupled with significant shifts in cellular pools of both nitrate and amino acids in general. Specifically, metabolite profiling of the OGDH antisense lines demonstrated that steady state levels of photorespiratory intermediates, namely glycerate, and glycine, were reduced, coupled with a significant reduction in the label redistribution to glycine and serine (Araújo et al., 2012b). These results suggest that the down-regulation of the TCA cycle activity was integrated with an up-regulation in the flux through the photorespiration pathway as part of a reprogramming to maintain either mitochondrial NADH homeostasis and/or the glutamate pool size. NADH and NADPH levels play an important role in mitochondrial respiratory metabolism and it can explain the maintenance of the levels of both NAD and NADPH in OGDH antisense plants (Araújo et al., 2012b). Altogether the results of this work coupled with others described above have clearly demonstrated that the alteration of the mitochondrial 2-OG metabolism has greater impacts in plant respiration and its connections than previously expected. It is important to mention that the precise nature of this interaction between 2-OG metabolism and hormone-mediated control of growth and senescence by GA remains an exciting topic for future research. For the purposes of this review perhaps most important is to outline the key role of 2-OG and by extension 2-ODD in GA metabolism.

**FIGURE 2:**
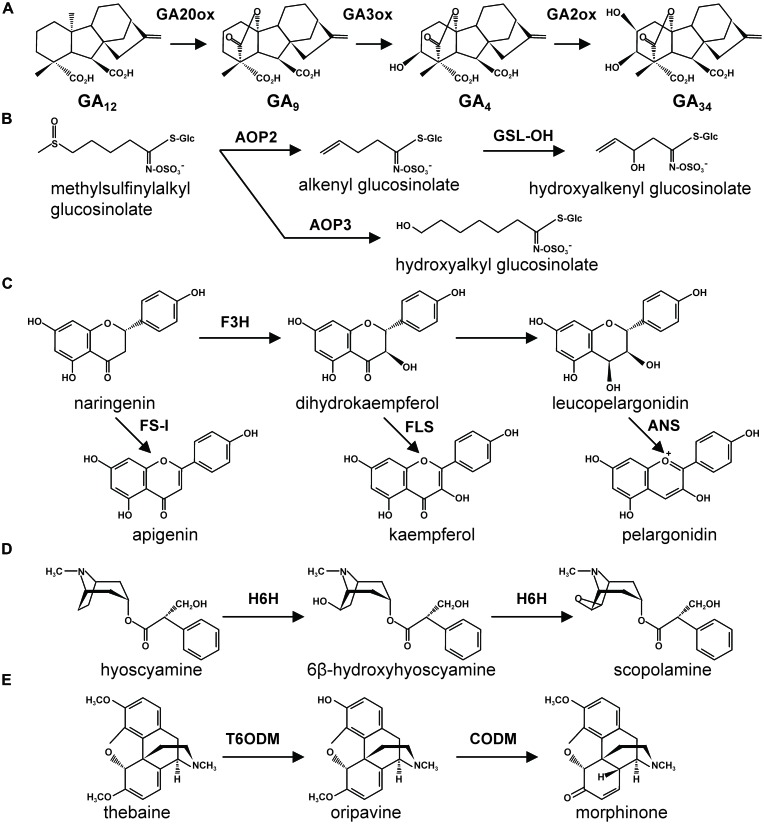
**Major enzymatic reactions of 2-ODD involved in GA and plant secondary metabolism.** 2-ODDs involved in **(A)** GA, **(B)** glucosinolate, **(C)** flavonoid, **(D)** tropan alkaloid, **(E)** isoquinorine biosynthesis are described. GA20ox, GA20-oxidase; GA3ox, GA3β-hydroxylase; GA2ox, GA 2-oxidase; AOP, alkenyl/hydroxy (OH) alkyl producing enzymes; GSL-OH, glucosinolate 2-oxoacid-dependant dioxygenase gene; FS-I, flavone synthase I; FLS, flavonol synthase, F3H, flavanone 3-hydroxylase, ANS, anthocyanin synthase; H6H, *hyoscyamine 6*-hydroxylase; T6ODM, 6-demethylase; CODM, codeine *O*-demehtylase.

It also presents metabolic evidence for a key role of 2-OG and 2-ODD in both GA metabolism. The synthetic enzymes involved in GAs biosynthesis have been well-characterized (Graebe, 1987). In GA biosynthesis, several types of oxidation enzymes are involved in GA biosynthesis, such as *ent*-kaurene oxidase (AtKO, CYP701A1) and *ent*-kaurenoic acid oxidase (AtKAO, CYP88A3, and CYP88A4) types, GA-β-hydroxylase and GA20-oxidase (2-ODD) types (Yamaguchi, 2008; Kawai et al., 2014). By great efforts of former works in GA metabolism, several 2-ODDs involved in GA biosynthesis have been found in several plant species (Prescott, 1993; **Figure 2A**); for example, 2β-hydoxylase(s) from *Phaseolus vulgaris* (Griggs et al., 1991) and from *Pisum sativum* (Smith and Macmillan, 1986), 2β-hydoxylase(s) from *P. vulgaris* (Smith et al., 1990), GA20-hydroxylase from *P. sativum* (Lange and Graebe, 1989) and from *Cucurbita maxima* (Lange and Graebe, 1989). Half of the reactions in GA biosynthesis are mainly converted by 2-ODDs (Hedden et al., 1982; Prescott, 1993). The identification and characterization of the 2-ODDs involved in GA biosynthesis has also led to major advances in the elucidation of the pathway including understanding of chemical diversity of GAs. Furthermore, it seems reasonable to consider that a part of GA biosynthesis is controlled by 2-OG content in plant.

## THE METABOLIC IMPORTANCE OF 2-ODD IN THE REGULATION OF SECONDARY METABOLISM

The oxygenase which leads to the incorporation of oxygen atoms from molecular oxygen is one of the most important enzymes in terms of conferring variation within plant secondary metabolism. Two functionally different classes of oxygenases, namely cytochrome P450 enzymes and 2-ODD, are involved in several pathways of plant secondary metabolism namely hydroxycinnamates, flavonoids and alkaloids (Tohge et al., 2013). As for GA biosynthesis, almost half of the oxidation reactions which are involved in late steps of their biosynthesis are catalyzed by 2-ODD (Tohge et al., 2013). In the biosynthesis of glucosinolates which are nitrogen/sulfur-containing secondary metabolites mostly found in *Brassica* species, three 2-ODDs, namely alkenyl /hydroxy (OH) alkyl producing enzymes (AOP1, AOP2, AOP3; Kliebenstein et al., 2001) and glucosinolate 2-oxoacid-dependent dioxygenase gene (GSL-OH; Hansen et al., 2008) have been found in *Arabidopsis* (**Figure 2B**). Since GSL-OH is evolutionarily highly distant from AOP2, it has been suggested that GSL-OH represents an independent recruitment from a different 2-ODD clade to the same metabolism (Kawai et al., 2014).

In flavonoid biosynthesis, four types of 2-ODDs; flavonol synthase (FLS), flavanone 3-hydroxylase (F3H), anthocyanin synthase (ANS also known as LDOX), and flavone synthase I (FS-I) are characterized as key enzymes in late steps of flavonoid aglycone formations resulting to in species specific flavonoid profiles (**Figure 2C**; Martens et al., 2001; Turnbull et al., 2004; Tohge et al., 2013). Flavonols are catalyzed from flavanone by hydroxylation of carbon-3 and 2,3-dehydration by F3H and FLS, respectively. On the other hand, ANS which catalyses the formation of dihydroflavonols from leucoanthocyanidins is known to be one of as one of the key enzymes involved in red pigmentation via anthocyanin and proanthocyanidin biosynthesis. FS-I which is found mainly in monocot and Apiaceae species uses flavanone as the substrate for flavone backbone formation. Given that F3H provides the precursor of substrates for FLS and ANS, it has been suggested that the evolution of FLS and ANS occurred after the emergence of F3H during 2-ODD evolution in seed plants (Stafford, 1991). Phylogenetic tree analysis using gene family of FLS, F3H, and ANS from several plant species revealed clear separation between FLS, F3H, and ANS as well as monocots, dicots and leguminous species as subclade of FLS, F3H, and ANS. This fact may suggest that FLS, F3H, and ANS have evolved in early stages of evolution (Tohge et al., 2013). In the other branches of phenolic secondary metabolism such as coumarin and 2,4-di-hydroxy-2*H*-1,4-benzoxazin-3(4*H*)-one (DIBOA) biosynthesis, several 2-ODDs have been found as key enzymes in their biosynthesis namely, *p*-coumaroyl-CoA 2′-hydroxylase (C2′H; Vialart et al., 2012), feruloyl-CoA 6′-hydroxylase (F6′H; Kai et al., 2008), and DIBOA-7-hydroxylase (BX6; Frey et al., 2003).

Several 2-ODDs are also involved in the synthesis of different alkaloids, which is the second largest class of plant secondary metabolism, have been characterized in several plant species. In the biosynthesis of tropane alkaloid, hyoscyamine 6-hydroxylase (H6H) in scoporamine biosynthesis of *Hyoscyamus niger* (**Figure 2D**; Matsuda et al., 1991), *Anisodus tanguticus* (Liu et al., 2005), and *Atropa belladonna* (Suzuki et al., 1999), 2′-deoxymugineic-acid 2′-dioxygenase (IDS3) in mugineic acid biosynthesis of *Hordeum vulgare* (Nakanishi et al., 2000) have been characterized. In addition, two 2-ODDs involved the biosynthesis of morphine namely thebaine 6-demethylase (T6ODM) and codeine *O*-demethylase (CODM) have been found form *Papaver somniferum* (**Figure 2E**; Hagel and Facchini, 2010).

## SUMMARY

In summary, 2-OG is not only a TCA cycle intermediate but also a co-factor for a diverse range of enzymes involved in amino acid, glucosinolate, flavonoid, alkaloid, and GA metabolism. Specifically in the case of GA and amino acid, recent evidence indicates that 2-OG levels control the rate of their biosynthesis. Further work is, however, needed to establish it also for glucosinolate, flavonoid, and alkaloid biosynthesis. Moreover, whilst preliminary studies have been attempted to understand evolutionary origins of the 2-ODD, more comprehensive analysis of this will be required to deepen our understanding of this important gene family.

## Conflict of Interest Statement

The authors declare that the research was conducted in the absence of any commercial or financial relationships that could be construed as a potential conflict of interest.
